# Robust, scalable and xeno-free protocol for differentiating human induced pluripotent stem cells into functional macrophages

**DOI:** 10.3389/fimmu.2025.1719452

**Published:** 2026-01-12

**Authors:** Miquel De Homdedeu, Rubén Escribá, Kenia Rodríguez-González, Sergi Querol, Jesus Fernandez-Sojo, Belén Alvarez-Palomo

**Affiliations:** 1 Cell Therapy Service, Banc de Sang i Teixits, Barcelona, Spain; 2 Transfusional medicine Group, Vall d’Hebron Research Institute (VHIR), Barcelona, Spain; 3 Josep Carreras Leukaemia Foundation, Barcelona, Spain

**Keywords:** allogenic cell therapy, gas-permeable rapid expansion system (G- Rex), good manufacturing practice (GMP), induced pluripotent stem cells (iPSCs), macrophage differentiation, phagocytosis

## Abstract

Human induced pluripotent stem cells (hiPSCs)-derived macrophages (iMacs) exhibit key macrophage phenotypic and functional properties, positioning them as promising candidates for allogenic cell immunotherapies. However, an efficient, scalable and good manufacturing practices (GMP)-compatible differentiation protocol is noticeably lacking. To meet this need, we aimed to develop a robust protocol for differentiating clinical-grade hiPSC lines into functional iMacs, designed for scalability and immediate GMP translation. We tested different media compositions, cytokine concentrations, seeding densities, culture regimens (2D vs. 3D) and coatings across key developmental stages. With the optimized protocol, we differentiated three different clinical-grade hiPSC lines towards mesoderm through embryoid body (EB) formation by agitation in 3D. Hematopoietic progenitor-producing EBs were induced to produce myeloid progenitors in agitation for 7 days. Then, myeloid progenitors were harvested and transferred to a G-Rex production platform to differentiate and further polarize into M1 or M2 iMacs. Cellular differentiation was assessed using flow cytometry panels through all developmental stages. At the final differentiated stage, iMacs were functionally characterized using the pHrodo phagocytosis assay. Pro-inflammatory cytokine secretion was analyzed by ELISA, and cell morphology was assessed by May-Grünwald Giemsa staining in Cytospin preparations. The full protocol was performed using feeder-free, scalable and either GMP or GMP- translatable reagents. This protocol enabled the production of iMacs with a mean purity of 98% viable cells, and a mean differentiation fold of 250X from a single hiPSC.

## 1 Introduction

Macrophages are central players in innate immunity, inflammation, and tissue homeostasis (1–3). In drug screening, macrophages act both as therapeutic targets and cellular readouts in treatments for cancer, autoimmune diseases, neurodegenerative disorders, and infections (4). In disease modeling and developmental studies, macrophages serve as a versatile tool to recapitulate pathological phenotypes and to study early embryonic processes such as organogenesis, angiogenesis, and tissue remodeling (5). Furthermore, their phagocytic ability, antigen-presenting activity and secretion of cytokines and chemokines make them very attractive in cell therapy (6–8).

Chimeric antigen receptor (CAR) therapy has emerged as an effective strategy in cancer immunotherapy. CAR-T cells have demonstrated remarkable efficacy in relapsed/refractory hematological malignancies (9), although some challenges such as cytokine release syndrome (CRS), immune effector cell-associated neurotoxicity syndrome (ICANS), graft-versus-host disease (GvHD), and donor heterogeneity remain to be addressed (9, 10). CAR-NK cells represent an alternative with advantages as reduced CRS and ICANS, lower risk of GvHD, and the potential for allogenic “off-the-shelf” therapies (11). In solid tumors, both CAR-T and CAR-NK face several obstacles including limited trafficking and infiltration, inactivation within the immunosuppressive tumor microenvironment (TME), off-target effects, and antigen escape (11). To overcome some of the limitations, efforts have focused on CAR-macrophages (CAR-Macs) (6, 7). Macrophages naturally infiltrate solid tumors, remodel the TME, and mediate phagocytosis, offering a unique advantage where CAR-T and CAR-NK therapies struggle. Beyond cancer, CAR-Macs have also shown potential for treating non-tumor diseases such as microbial and viral infections, Alzheimer’s disease, fibrosis, and atherosclerosis (12).

Considering these applications, large numbers of macrophages of consistent quality are required. However, access to primary human macrophages is limited by donor variability, low cell numbers, and minimal self-renewal capacity (13). In this context, human induced pluripotent stem cells (hiPSCs), generated by reprogramming somatic cells with the Yamanaka factors (Oct3/4, Sox2, Klf4, and c-Myc) (14), represent an attractive cell source for macrophage-based therapies owing to their unlimited proliferation and differentiation potential (7). Furthermore, the use of HLA-homozygous hiPSCs enables the production of highly immunocompatible allogenic cell therapies that can avoid host rejection and extend cell therapy products persistence (15). Moreover, HLA compatibility would enable the antigen presenting function of macrophages leading to a stronger immune response.

iPSCs have been reported to be a good source of functional macrophages (6–8, 16–18). Although the first protocols to differentiate hiPSCs into macrophages were based on monolayer (2D) culture systems (19), the need for large-scale production has favored the implementation of embryoid body (EB)-based (3D) strategies that can be adapted to production in bioreactors (16, 17, 20). Compared to 2D cell cultures, 3D protocols require fewer exogenous growth factors and enable more physiological differentiation by recapitulating early embryogenesis, providing a niche for mesoderm induction, hematopoietic specification, and myeloid lineage commitment within the EB structure (21). Combined with bioreactors, these approaches offer the potential for higher yields, highlighting the need for alternative protocols that combine the physiological advantages of 3D differentiation with scalability and reproducibility (22). However, several drawbacks remain, including fluid shear stress in stirred-tank bioreactors, limited oxygen and nutrient diffusion within dense 3D cultures, the need for further process optimization to achieve relevant yields, and the requirement for highly specialized training and equipment (17). To address these limitations, we describe a robust, scalable, and xeno-free protocol workflow for the efficient differentiation of hiPSCs into functional macrophages, combining 3D cell culture with the Gas-permeable Rapid Expansion (G-Rex) system (23). We outline the key optimization steps, assess the phenotypic and functional integrity of the resulting cells, and demonstrate the suitability of this protocol for both research and clinical contexts.

## 2 Materials and methods

### 2.1 Human induced pluripotent stem cell cultivation

Experiments were performed with three different clinical-grade HLA-homozygous hiPSC lines from the iPS-PANIA iPSC cell line collection (ID ethics committee: PR(AG)428/2018): Hz 1-8-3 CBiPS4-Sv4F-F6 (HLA-A 01:01, HLA-B 08:01, HLA-DRB1 03:01), Hz 33-14-1 CBiPS6-Sv4F-H6 (HLA-A 33:01, HLA-B 14:02, HLA-DRB1 15:01), and Hz 24-7-15 CBiPS7-Sv4F-I12 (HLA-A 24:02, HLA-B 07:02, HLA-DRB1 15:01) (24). hiPSCs were expanded in feeder-free monolayer culture on Vitronectin (Gibco Cat#A31804) coated 6-well plates using TeSR™-AOF (Stemcell Technologies Cat#100-0401) in an incubator at 37°C with 5% CO2. Passages of colonies were performed using CTS™ Versene™ (Gibco Cat#A4239101) every 3–4 days, according to the manufacturer’s instructions.

### 2.2 Differentiation of iPSCs to hematopoietic stem cells

hiPSCs were cultured in TeSR™-AOF supplemented with Rock inhibitor Y27632 (10 µM; Sigma-Aldrich Cat#Y0503) at least 30min before the differentiation. Undifferentiated hiPSCs were harvested using Accutase™ (Thermo Fisher Scientific Cat#A1110501), counted, and 65×10^3^ single-cell iPSCs were transferred per well into non-adherent 12-well culture plates (Corning Cat#3737) to allow formation of embryoid bodies (EBs) in mesoderm induction medium made of STEMdiff™ APEL™2 medium (Stemcell Technologies Cat#5270) supplemented with BMP-4 (20 ng/mL; R&D Systems Cat#314-BP-010/CF), VEGF (200 ng/mL; Stemcell Technologies Cat#78159.1), bFGF (200 ng/mL; Stemcell Technologies Cat#78134.1), Activin A (100 ng/mL; R&D Systems Cat#338-BP-010), and 10 µM Rock inhibitor Y27632 during 3 days with orbital rotation at 80 rpm. At day 3, half of the medium was replaced with hematopoietic induction medium made of SFM StemPro™-34 (Thermo Fisher Scientific Cat#10639011) supplemented with Glutamax (1X Gibco Cat#35050038), non-essential amino acids (NEAA; 1X Gibco Cat#11140050), insulin-transferrin-selenium-ethanolamine (ITS; 1X Gibco Cat#51500056), 2-mercaptoethanol (BME; 50 µM Gibco Cat#31350010), 10 ng/mL BMP-4, 200 ng/mL VEGF, 50 ng/mL bFGF, SCF (100 ng/mL; Thermo Fisher Scientific Cat#AF-300-07), FLT3L (20 ng/mL; Thermo Fisher Scientific Cat#AF-300-19), IL-3 (20 ng/mL; Miltenyi Biotec Cat#130-095-070) and L-Ascorbic acid (50 ng/mL; Sigma-Aldrich Cat#A4403-100MG). On days 5 and 7, complete and half medium changes were performed, respectively, with hematopoietic induction medium. Agitation was maintained until day 10. Then, secreted hematopoietic stem cells (HSCs) were counted and analyzed using the hematopoietic stem cells flow cytometry panel, as described below.

### 2.3 Optimization of differentiation of hematopoietic stem cells into myeloid progenitors

Differentiation of HSCs into myeloid progenitors (MPs) was optimized using 2D and 3D approaches. In 2D, differentiation was performed seeding 25x10^3^ CD34^+^ cells either onto a 24mw tissue culture-treated well plate (Sarstedt Cat#83.3922) or onto a 24mw G-Rex plate (Wilson Wolf Cat#80192M). 24mw tissue culture-treated well plates were coated with 500 µL of Matrigel (200-277.5 µg/mL; Corning Cat#356234) diluted 1/40 in Opti-MEM (Gibco Cat#31985070), 0.1% gelatin (Sigma-Aldrich Cat#G1890-100G) or laminin-521 (Stemcell Technologies Cat#200-0117). During 7 days (from day 10 to day 17) CD34^+^ HSCs cells were derived to MPs in myeloid induction medium made of SFM StemPro™-34 supplemented with 1X Glutamax, 1X NEAA, 1X ITS, 50 µM BME, 5% human AB serum (hAB; clinical grade, pool of 3 to 6 donors, manufactured by The Blood and Tissue Bank (Banc de Sang i Teixits, Barcelona, Spain) Cat#D5144V00), M-CSF (100 ng/mL; PeproTech Cat#300-25-50UG), 25 ng/mL IL-3, and 50 ng/mL L-Ascorbic acid. Cells were cultured in 24mw tissue culture-treated plates with 500 µL per well. On days 12 and 14, 250 µL of fresh myeloid induction medium were added per well. 24mw G-Rex plates were left uncoated, filled with 4 mL of myeloid induction medium per well, and seeded with hematopoietic progenitor-producing EBs together with CD34^+^ cells. A spike-in of total M-CSF, IL-3 and L-Ascorbic acid was also performed at day 14. In 3D, 6mw non-adherent well plates (Sarstedt Cat#83.3920.500) were seeded with a single or multiple EBs with 25, 50 or 75x10^3^ CD34^+^ cells per well, in 2 mL of myeloid induction medium with rotation at 80 rpm. On days 12 and 14, 1 mL of fresh myeloid induction medium was added per well.

GM-CSF (PeproTech Cat#300-03-20UG) was tested as a partial or full replacement for M-CSF in myeloid induction medium by comparing conditions with 100 ng/mL GM-CSF, 50 ng/mL GM-CSF and 50 ng/mL M-CSF, and 100 ng/mL M-CSF. On day 17, differentiated MPs were counted and analyzed using the myeloid progenitor cells flow cytometry panel, as described below.

### 2.4 Optimization of differentiation of myeloid progenitors into M0 macrophages

Differentiation of MPs into hiPSCs-derived macrophages (iMacs) was optimized using 2D and 3D approaches. In 2D, differentiation was performed by seeding MPs at a wide range of densities onto 24mw tissue culture plates that were either left uncoated or coated with 500 µL of 200-277.5 µg/mL Matrigel diluted in Opti-MEM, 0.1% gelatin, or laminin. Over a 7-day period (from day 17 to day 24) MPs were differentiated into iMacs in macrophage induction medium consisting of SFM StemPro™-34 supplemented with 1X Glutamax, 1X NEAA, 1X ITS, 50 µM BME, 5% hAB serum, and 100 ng/mL M-CSF. Cultures were maintained in 24mw plates with 500 µL of macrophage induction medium per well. On days 19 and 21, 250 µL of fresh macrophage induction medium were added per well. In this stage, cells were cultured with 100 ng/mL GM-CSF, 50 ng/mL GM-CSF and 50 ng/mL M-CSF, or 100 ng/mL M-CSF. 24mw G-Rex plates were left uncoated, filled with 4 mL of macrophage induction medium per well, and seeded with different densities of MPs. None, one, two or three M-CSF spike-in regimens were performed at day 21; days 19 and 21; or days 19, 21 and 23, respectively.

In 3D, 6mw non-adherent well plates were seeded with different densities of MPs per well, in 2 mL of macrophage induction medium with rotation at 80 rpm. On days 19 and 21, 1 mL of fresh macrophage induction medium was added per well. On day 24, differentiated M0 iMacs were counted and analyzed using the iMacs flow cytometry panel, as described below.

### 2.5 Polarization of M0 macrophages towards M1 or M2 phenotype

M0 iMacs were polarized towards either M1 or M2 phenotype by culturing them in M1 or M2 polarization medium, consisting of macrophage induction medium supplemented with 100 ng/mL LPS (Sigma-Aldrich Cat# L4391-1MG) and 20 ng/mL IFN-γ (PeproTech Cat# 300-02 100ug), or 20 ng/mL IL-4 (PeproTech Cat#200-04-5UG) and 20 ng/mL IL-13 (PeproTech Cat#200-13-2UG), respectively. iMacs were maintained in 24mw tissue culture plates or 24mw G-Rex plates, with 500 µL or 4 mL of M1 or M2 polarization medium per well, respectively. On days 26 and 28, 250 µL of fresh M1 or M2 polarization medium were added per well for 24mw tissue culture plate polarizations. On day 28, a cytokine spike-in was performed in the 24mw G-Rex plate polarizations. On day 31, polarized M1 or M2 iMacs were counted and analyzed using the iMacs flow cytometry panel, as described below.

### 2.6 Flow cytometry analyses

Flow cytometry analyses were performed at different stages of cellular differentiation, using the panels described in **Table 1**. Secreted hematopoietic stem cells were analyzed for the expression of CD34, CD45, and CD43; myeloid progenitor cells for CD14, CD16, and CD45; and M0, M1, and M2 iMacs for CD14, CD86, and CD206.

**Table 1:** Flow cytometry panels.

Panel	Reagent	Source	Catalogue number
Hematopoietic stem cells	ViaKrome 405 Fixable Viability Dye	Beckman Coulter	C36614
	CD34 PE	Beckman Coulter	A07776
	CD45 FITC	Beckman Coulter	A07782
	CD43 APC A750	Beckman Coulter	B49195
Myeloid progenitor cells	ViaKrome 405 Fixable Viability Dye	Beckman Coulter	C36614
	CD14 ECD	Beckman Coulter	B92391
	CD16 KrO	Beckman Coulter	B00069
	CD45 FITC	Beckman Coulter	A07782
iMacs	ViaKrome 405 Fixable Viability Dye	Beckman Coulter	C36614
	CD14 ECD	Beckman Coulter	B92391
	CD86 APC A750	Beckman Coulter	B30646
	CD206 PE	Beckman Coulter	IM2741

iMacs, human induced pluripotent stem cell-derived macrophages.

Myeloid progenitor cells and iMacs were stained after blocking non-specific Fc receptor binding with Fc Block (BD Biosciences Cat#564220). All samples were mixed with Flow-Count Fluorospheres (Beckman Coulter Cat#7547053), washed, and acquired in FACS buffer. Sample acquisition was performed using DxFLEX Flow Cytometer (Beckman Coulter) and analyzed with Kaluza Analysis Software (Beckman Coulter) and FlowJo software (Tree Star Inc.). The gating strategy for all panels included: exclusion of cellular debris using Forward Scatter (FSC-A) and Side Scatter (SSC-A), selection of single cells using FSC-A and FSC-H, and identification of live cells using SSC-A and ViaKrome 405 (Beckman Coulter Cat#C36614). Resulting iMacs were further analyzed for HLA-DR expression using HLA-DR APC (Beckman Coulter Cat#IM3635). All cellular populations were quantified using Flow-Count Fluorospheres according to the manufacturer’s instructions.

### 2.7 Cytospin preparation

A total of 50,000 M0 iMacs were spun on glass slides at 5000×g 10 min (Cytospin4, Shandon, Thermo Scientific), air-dried, and stained for 5 min in May-Grünwald and 10 min in 30% Giemsa (Sigma). Images were taken under optical microscopy (Leica DFC450 C).

### 2.8 Cytokine production assays

GM-CSF, Granzyme-B, IFN-γ, IL-2, IL-4, IL-6, IL-10, IL-17A, IL-21, MCP-1 (CCL2), Perforin, and TNF levels from M0 iMacs supernatant were assessed using MACSPlex Cytotoxic T/NK Cell Kit (Miltenyi Biotec Cat#130-125-800) following manufacturer’s instructions. Sample acquisition was performed using MACSQuant Analyzer 10 Flow Cytometer (Miltenyi Biotec), and data analysis with MACSPlex InspectoR tool (Miltenyi Biotec). Samples were analyzed in duplicate.

### 2.9 Phagocytosis assay

M0, M1, and M2 iMacs were co-cultured with pHrodo Deep Red E. coli BioParticles (Invitrogen Cat#P35360) in DMEM FluoroBrite (Gibco Cat#A1896701), according to the manufacturer’s instructions. After 2h 30min at 37°C, co-cultures were imaged under fluorescence microscopy (Leica DFC450 C). Processing of images was conducted through ImageJ software. Samples were also characterized by flow cytometry, acquired using DxFLEX Flow Cytometer and analyzed with Kaluza Analysis Software and FlowJo.

### 2.10 Statistical analysis

Statistical analysis and preparation of graphs were performed using Prism version 5.03 (GraphPad Software). For comparisons between two groups, the Mann-Whitney test was used. For comparisons among more than two groups, the Kruskal-Wallis test with Dunn’s post-test was applied. A two-tailed *p*-value below 0.05 was considered statistically significant.

## 3 Results

Our aim was to optimize the production of M1 polarized macrophages from iPSC with a protocol that could be easily translatable to an up-scaled and GMP grade production for clinical studies. We focused on two aspects: (i) obtaining a feeder-free protocol using xeno-free media and reagents that are also available as a GMP version (**Supplementary Table 1**). (ii) Adapting each stage to either growth in suspension to make it up-scalable to 3D agitation bioreactors or in adhesion on non-coated surfaces to make it up-scalable to G-Rex type bioreactors. Moreover, we identified the optimal seeding densities to obtain the highest expansion fold with the minimal culture surface and media volume, for a feasible and efficient future manufacturing process.

### 3.1 Differentiation of iPSCs into hematopoietic stem cells

**Figure 1:**
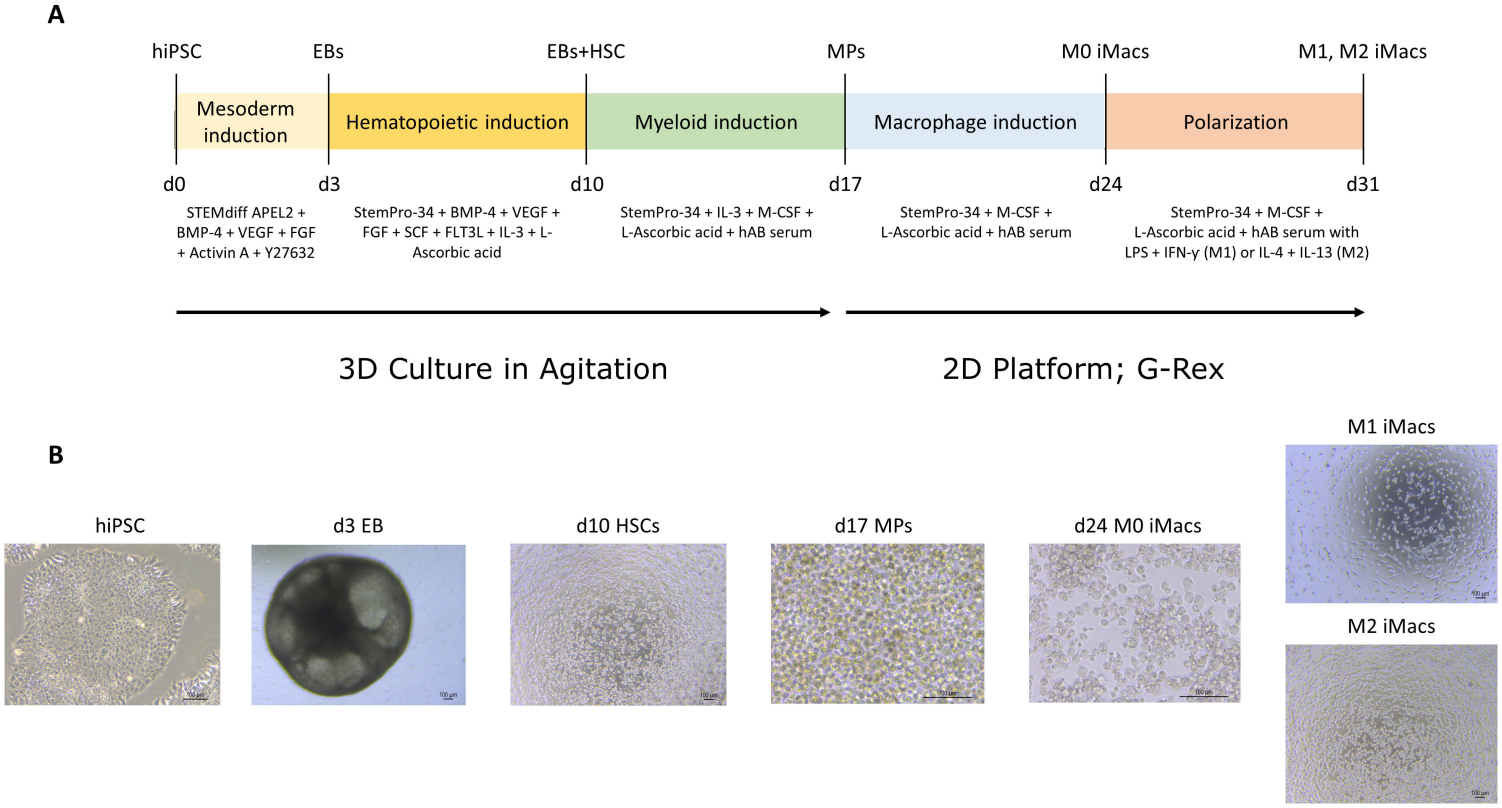
Differentiation of hiPSC into M0, M1 and M2 polarized iMacs. **(A)** Scheme of differentiation of hiPSC into M0, M1 and M2 polarized iMacs. **(B)** Representative brightfield images showing the different cellular stages: hiPSCs (adhered, compact colonies, with a high nucleus-to-cytoplasm ratio, and no signs of spontaneous differentiation), EBs (at day 3 they measure >300µm, have spherical morphology and show clear boundaries), HSCs (small and very bright round cells), MPs (slightly bigger and very bright round cells), M0 iMacs (large, foamy, and cells with a vacuolated cytoplasm), and M1(Elongated, spindle-shaped cells)- and M2(large, round, and flat-shaped cells with an amoeboid appearance)-polarized iMacs. Images were acquired at x40-x200 magnifications, scale bar represents 100 µm. EB, embryoid Body; HSC, hematopoietic stem cell; MP, myeloid progenitor; iMacs, hiPSCs-derived macrophages; d, day.

**Figure 2:**
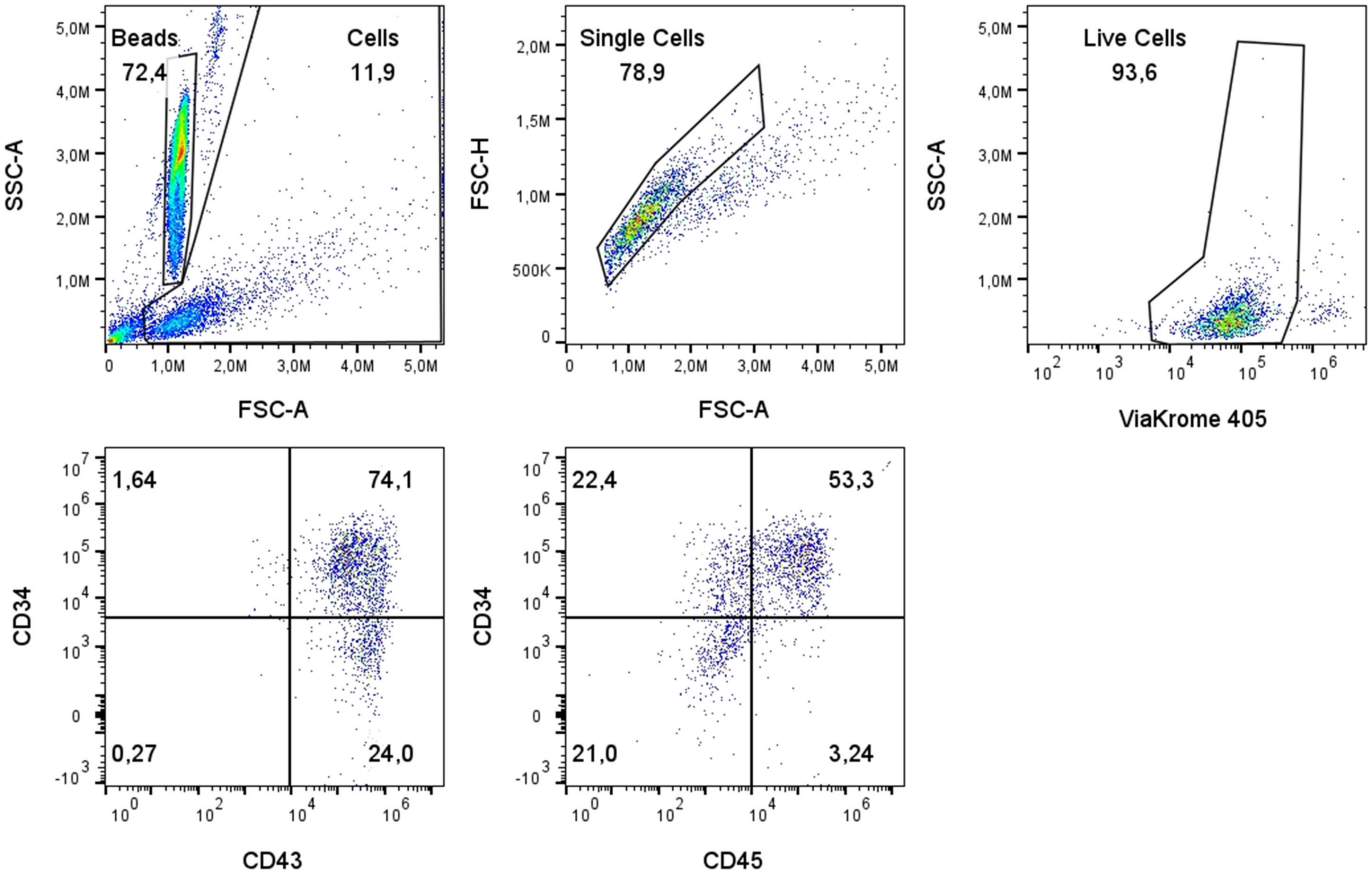
Characterization of secreted hematopoietic stem cells. Representative plots of the gating strategy used during flow cytometry analysis: exclusion of cellular debris using Forward Scatter (FSC-A) and Side Scatter (SSC-A); selection of single cells using FSC-A and FSC-H; identification of live cells using SSC-A and ViaKrome 405; scatter dot plot showing the expression of CD34 and CD43; and scatter dot plot showing the expression of CD34 and CD45.

hiPSCs were successfully differentiated into HSCs using the EB model in a two-step process consisting of mesoderm induction followed by hemogenic induction (**Figure 1A**). From 65×10^3^ single-cell hiPSCs, between 1 to 3 EBs were formed in mesoderm induction medium over three days (**Figure 1B**). After an additional seven days in hemogenic induction medium, EBs yielded an average of 1.2x10^6^ live single cells, 75% being CD34^+^CD43^+^ and 55% CD34^+^CD45^+^, consistent with a hematopoietic phenotype (**Figure 2**).

### 3.2 Optimization of differentiation of hematopoietic stem cells into myeloid progenitors

**Figure 3:**
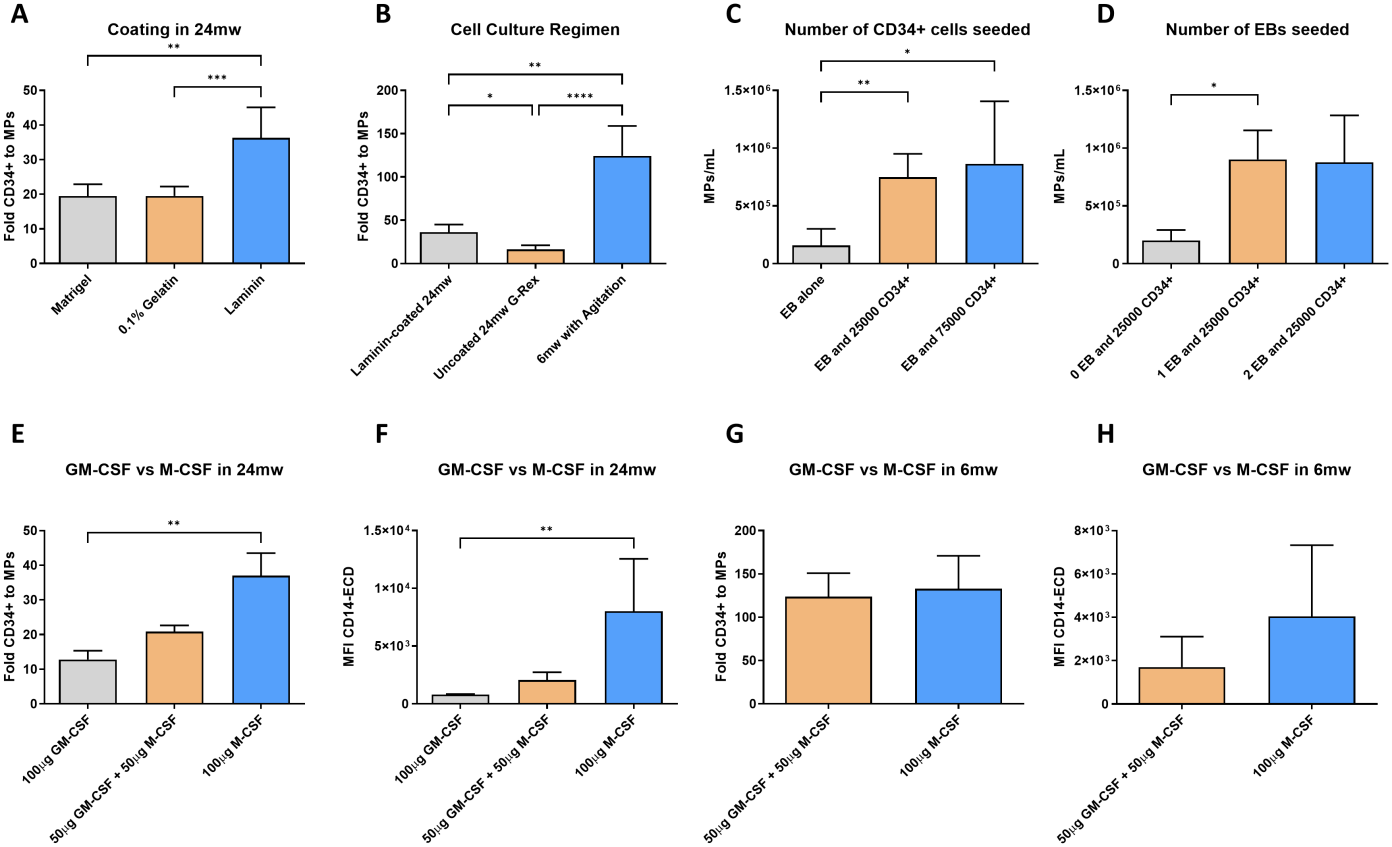
Optimization of differentiation of hematopoietic stem cells into myeloid progenitors. Bar plots comparing different strategies for optimizing the HSC-to-MP differentiation stage. **(A)** Fold change of CD34^+^ to MPs relative to 24mw Coating. **(B)** Fold change of CD34^+^ to MPs relative to cell culture regimen. **(C)** Density of differentiated MPs generated by seeding a single EB with different amounts of CD34^+^ HSCs. **(D)** Density of differentiated MPs generated by seeding different amount of EBs with the same amount of CD34^+^ HSCs. **(E)** Fold change of CD34^+^ to MPs relative to partial or full replacement of M-CSF by GM-CSF in 24mw plates. **(F)** Expression of CD14 in the differentiated MP population relative to partial or full replacement of M-CSF by GM-CSF in 24mw plates. **(G)** Fold change of CD34^+^ to MPs relative to partial replacement of M-CSF by GM-CSF in 6mw plates. **(H)** Expression of CD14 in the differentiated MP population relative to partial replacement of M-CSF by GM-CSF in 6mw plates. (n=4-13; *P < 0.05, **P < 0.01, ***P < 0.001; Mann-Whitney and Kruskal-Wallis test with Dunn’s post-test; mean ± SD).

Following the generation of HSCs from hiPSCs, we optimized their differentiation into myeloid progenitors (MPs) using a 2D culture system (**Figures 1A, B**). The 2D differentiation process on standard tissue culture-treated plates was optimized and used as a reference (**Figure 3**). In 24-well culture plates, 25×10^3^ HSCs per well were identified as the optimal seeding density based on previous experiments performed in our laboratory. In order to identify the best coating to differentiate these HSCs into myeloid progenitors in a 24mw cell culture, we compared Matrigel, 0.1% gelatin, and laminin 521 (**Figure 3A**). At day 17, laminin coating induced the higher production of MPs by number of HSCs seeded compared with Matrigel, and 0.1% gelatin coatings (mean fold of 19.49, 19.52 and 36.26; *p* < 0.01 and *p* < 0.001, respectively).

Once laminin coating was identified as the best option tested to differentiate CD34^+^ HSCs to CD45^+^CD14^+^ MPs in 24mw cell culture plates, to test bioreactor-scalable approaches, we compared this strategy against using a 24mw G-Rex and a 6mw non-adherent well plate seeding hematopoietic progenitor-producing EBs together with 25x10^3^ CD34^+^ cells (**Figure 3B**). We observed that seeding hematopoietic progenitor-producing EBs in 6mw under rotation induced the higher production of MPs by number of CD34^+^ HSCs seeded compared with laminin coated 24mw, and 24mw G-Rex (mean fold of 36.26, 16.18, and 124.4; *p* < 0.05, *p* < 0.01 and *p* < 0.001, respectively). Observing that seeding HSC-producing EBs together with CD34^+^ HSCs produced the major amount of CD45^+^CD14^+^ MPs, we studied different combinations of EBs and CD34^+^ HSCs cells. When comparing the effect of seeding a single EB with none, 25x10^3^ or 75x10^3^ CD34^+^ cells, we observed statistically significant differences seeding 25x10^3^ and 75x10^3^ HSCs compared against the EB alone (*p* < 0.01 and *p* < 0.05, **Figure 3C**). Surprisingly, a stronger statistical difference was observed when seeding the EB with 25x10^3^ HSCs, as this condition was more robust, whereas seeding the EB with 75x10^3^ HSCs showed greater variability. Therefore, seeding a single EB together with 25x10^3^ HSCs was identified as the optimal combination. When comparing the effect of seeding multiple EBs with 25x10^3^ CD34^+^ HSCs – corresponding to multiple 12mw wells-, statistical significance was only observed between the absence and the presence of 1 EB, with no significant differences between seeding one or two (*p* < 0.05, **Figure 3D**).

Next, to maximize proliferation and differentiation of myeloid progenitors, we tested different variations of the myeloid medium, partially or fully replacing M-CSF by GM-CSF. In 24mw plates, we observed a lower fold and a lower expression of CD14 when using GM-CSF instead of M-CSF in the myeloid medium (*p* < 0.01 and *p* < 0.01, **Figures 3E, F**). Although not significant, in rotation in 6mw plates, the same reduction was observed (**Figures 3G, H**). Taken together, these results show that differentiation of hematopoietic stem cells into myeloid progenitors is more efficient when performed in a 3D culture, here done in a 6mw, seeding an EB with 25x10^3^CD34^+^ HSCs in myeloid induction medium containing M-CSF as the main cytokine during the seven-days stage.

### 3.3 Optimization of differentiation of myeloid progenitors into M0 macrophages

**Figure 4:**
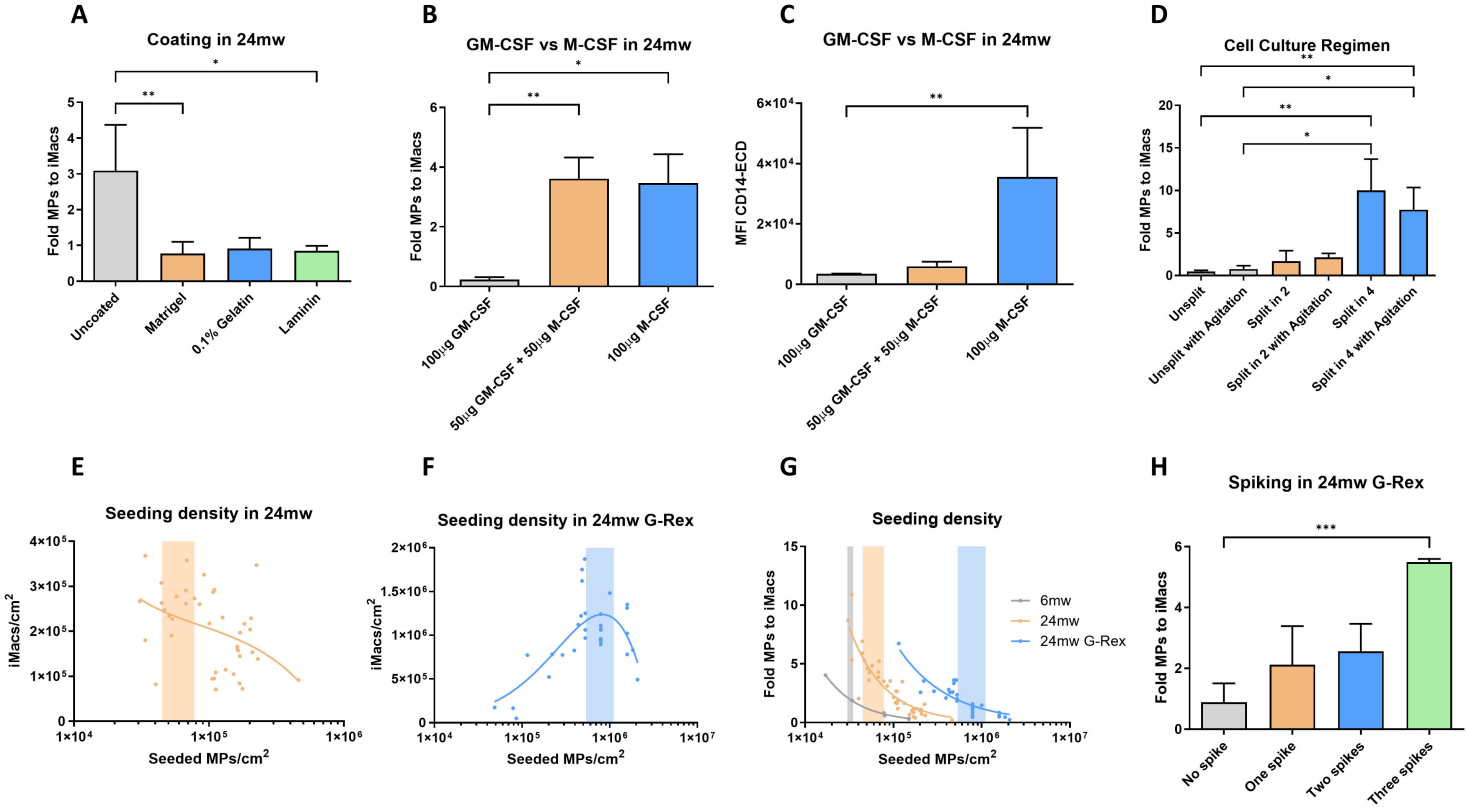
Optimization of differentiation of myeloid progenitors into macrophages. Bar plots and fold-change curves comparing different strategies for optimizing the MP-to-iMacs differentiation stage. **(A)** Fold change of MPs to iMacs relative to 24mw Coating. **(B)** Fold change of MPs to iMacs relative to partial or full replacement of M-CSF by GM-CSF in 24mw plates. **(C)** Expression of CD14 in the differentiated iMacs population relative to partial or full replacement of M-CSF by GM-CSF in 24mw plates. **(D)** Fold change of MPs to iMacs relative to cell culture regimen. **(E)** Density of differentiated iMacs obtained by seeding MPs at varying densities in 24mw plates. The colored range indicates the optimal seeding window. of MPs. **(F)** Density of differentiated iMacs obtained by seeding MPs at varying densities in 24mw G-Rex. The colored range indicates the optimal seeding window. of MPs. **(G)** Fold-change curves of MP-to-iMac differentiation as a function of MP seeding density in 6mw, 24mw, and 24mw G-Rex plates. The colored range indicates the optimal seeding window. of MPs. **(H)** Fold change of MPs to iMacs relative to number of M-CSF spiking in 24mw G-Rex. (n=4-31; *P < 0.05, **P < 0.01, ***P < 0.001; Mann-Whitney and Kruskal-Wallis test with Dunn’s post-test; mean ± SD).

Following the optimization of differentiation of CD34^+^ HSCs into MPs, we next proceed with the MP-to-iMac step (**Figures 1A, B**). The 2D differentiation process on standard tissue culture-treated plates was optimized and used as a reference when comparing with other culture regimens (**Figure 4**). In order to identify the best coating to differentiate MPs into iMacs, we compared differentiation fold between uncoated wells and coated with Matrigel, 0.1% gelatin, and laminin. In this stage, the absence of coating resulted in a higher differentiation fold compared with Matrigel, 0.1% gelatin, and laminin (mean fold of 3.09, 0.77, 0.91, and 0.84, respectively; **Figure 4A**), with statistical significance observed when compared to Matrigel and laminin coatings (*p* < 0.01 and *p* < 0.05, respectively). Next, we tested different variations of the macrophage medium, partially or fully replacing M-CSF by GM-CSF. In 24mw plates, we observed a lower fold and a lower expression of CD14 when using GM-CSF instead of M-CSF (*p* < 0.05 and *p* < 0.01, **Figures 4B, C**).

In order to identify the best cell culture regimen to differentiate MPs into iMacs, we analyzed the effect of culturing them in rotation and the effect of MP seeding density. Agitation had no beneficial effect on iMacs differentiation. However, significant differences were found when comparing splitting 1 in 4 the MP before induction of differentiation to macrophages (*p* < 0.01; **Figure 4D**).

As seeding density appeared to be crucial in the MP-to-iMac differentiation step, we tested a wide range of seeding densities in 24mw culture plates and 24mw G-Rex plates, and plotted differentiated iMacs/cm^2^ against the initial seeded MPs/cm^2^. Surprisingly, an exponential decrease in the yield of differentiated iMacs was observed with increasing densities of seeded MPs in 24mw culture plates, identifying the range of maximal efficiency between 4.5x10^4^ and 7.8x10^4^ MPs/cm^2^ (**Figure 4E**). In 24mw G-Rex culture system, differentiation followed a non-linear regression model (**Figure 4F**), with the formula:


$$Y=\frac{DiffMax\times X}{Kd+X}+(NS\times X)$$

Where Y represented density (n/cm^2^) iMacs differentiated, X the density of seeded MPs, DiffMax total differentiated iMacs, Kd the efficiency differentiation constant, and NS for the slope of the nonlinear regression. DiffMax had a value of 3.17x10^6^, Kd was 495891, and NS was -0.9037. With this formula, 8,24x10^5^ MPs/cm^2^ was identified as the most efficient seeding density, with a ±30% range (5,35x10^5^ to 1,11x10^6^ MPs/cm^2^) defined as the optimal seeding window. When comparing MP-to-iMac differentiation fold between 6mw, 24mw culture plates and 24mw G-Rex culture system, the G-Rex platform significantly outperformed standard cell culture plates, allowing a similar yield with a 10 times higher initial cellular density (**Figure 4G**). Finally, we analyzed the effect of total cytokine spiking in 24mw G-Rex culture system on MP-to-iMacs. Notably, performing three M-CSF spikes over the seven-day stage significantly increased yield when compared with no spiking (*p* < 0.001; **Figure 4H**).

Taken together, these results show that differentiation of myeloid progenitors into macrophages is more efficient when performed in a 2D culture, in a 24mw G-Rex, seeding between 4.8x10^5^ and 10^6^ MPs/cm^2^ in macrophage induction medium containing M-CSF, and performing 3 spikes of total M-CSF during the seven-days stage.

### 3.4 Characterization of M0 iMacs.

**Figure 5:**
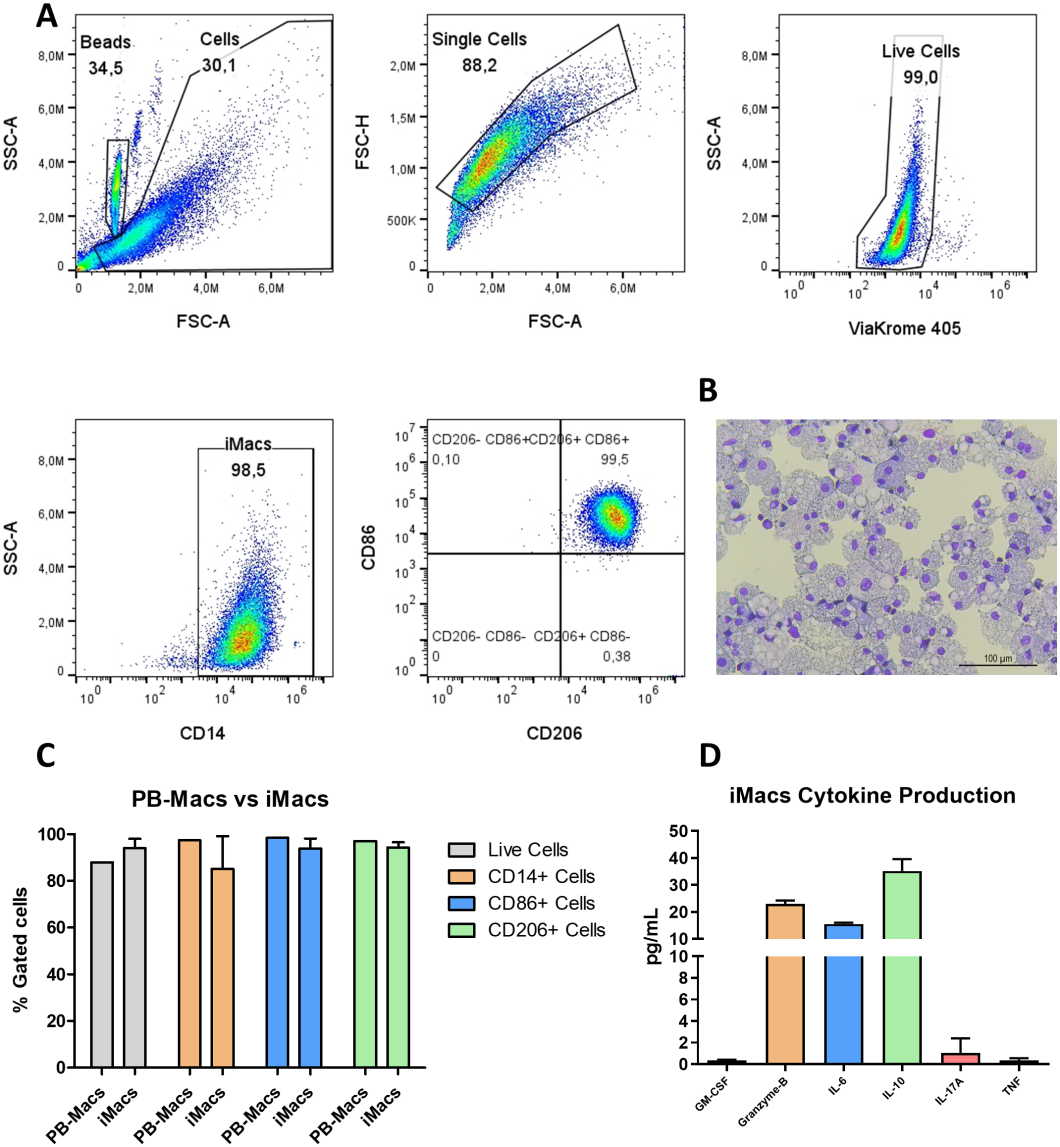
Phenotypic and functional characterization of M0 iMacs. **(A)** Representative plots of the gating strategy used during flow cytometry analysis: exclusion of cellular debris using FSC-A and SSC-A; selection of single cells using FSC-A and FSC-H; identification of live cells using SSC-A and ViaKrome 405; identification of iMacs using SSC-A and CD14; and scatter dot plot showing the expression of CD86 and CD206. **(B)** Representative brightfield Cytospin staining for M0 iMacs. **(C)** Comparison of Live Cells, and CD14, CD86, and CD206 expression between macrophages derived from primary peripheral blood monocytes and iPSC-derived macrophages. (PB-Macs: n=1 used as reference; iMacs: n=3; data shown as mean ± SD.) **(D)** Cytokine levels produced by iMacs: GM-CSF, Granzyme-B, IL-6, IL-10, IL-17A, and TNF. Images were acquired tat ×200 magnification, scale bar represents 100 µm. (n=2; mean ± SD).

Final hiPSC-derived M0 macrophages were assayed for phenotype and functional characterization. At the end of macrophage induction, 98% of the cells expressed CD14, CD86, and CD206; the expression of this markers was similar to macrophages derived from primary peripheral blood monocytes (**Figure 5C**, **Supplementary Figure 1**). In May-Grünwald Giemsa staining, M0 iMacs appeared as large cells measuring around 50 µm, with abundant foamy, pale blue cytoplasm and a small purple-stained nucleus (**Figure 5B**). When analyzing cytokine production in iMacs supernatants, we observed levels of GM-CSF, Granzyme-B, IL-6, IL-10, IL-17A and TNF, indicating a high capacity to induce inflammatory signaling and monocyte attraction (**Figure 5C**).

### 3.5 Polarization and characterization of M1 and M2 iMacs.

**Figure 6:**
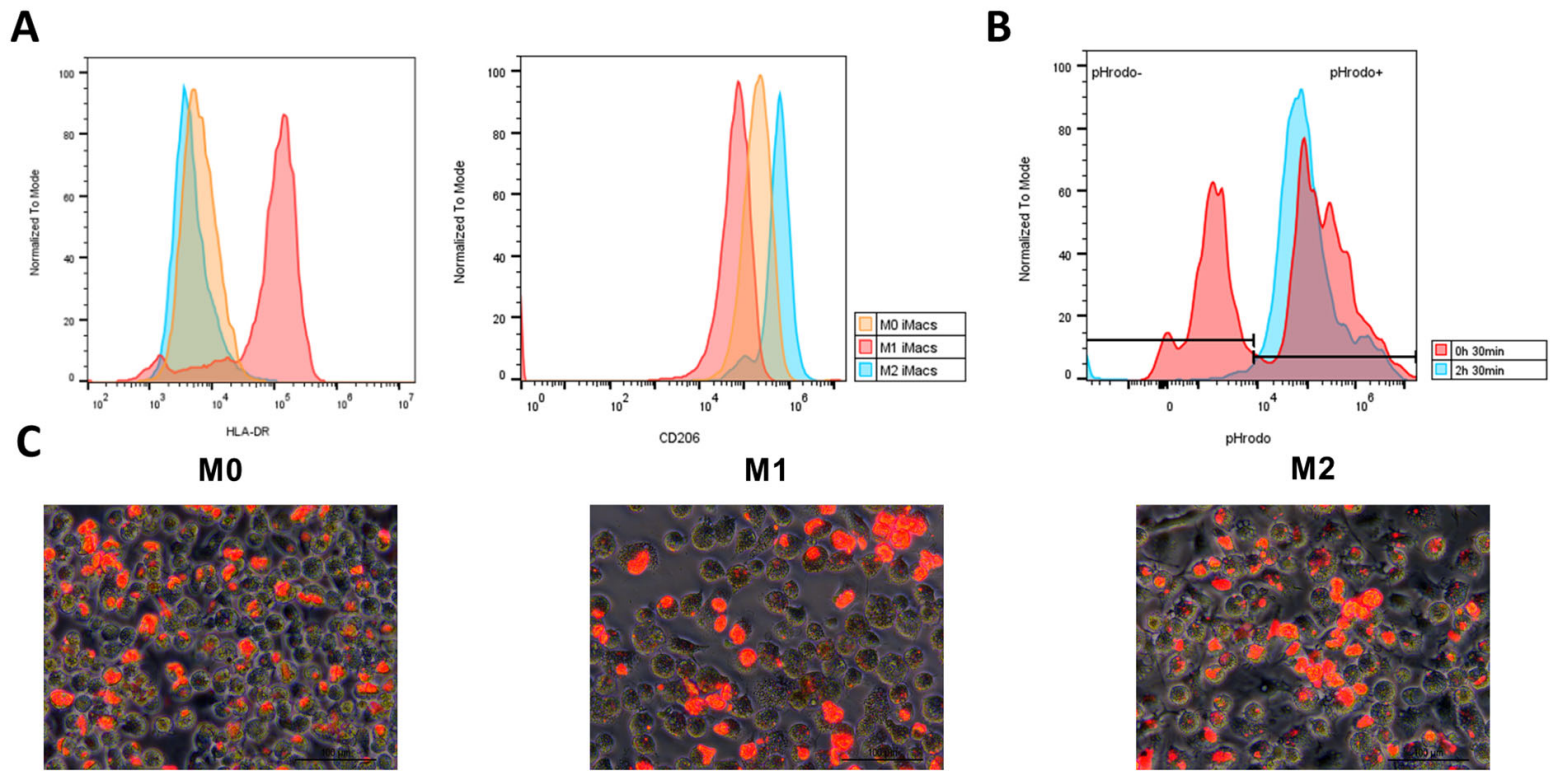
Phenotypic and functional characterization of M0, M1 and M2 iMacs. **(A)** Overlay of MFI histograms comparing HLA-DR and CD206 expression between M0, M1 and M2 iMacs. **(B)** Overlay of MFI histograms comparing pHrodo expression in M1 iMacs after incubation with Deep Red E. coli BioParticles for 30min or 2h 30min at 37°C. **(C)** Fluorescence microscopy images of M0, M1, and M2 iMacs after internalization of pHrodo E. coli BioParticles for 2h 30min at 37°C. Images were acquired tat ×200 magnification, scale bar represents 100 µm.

hiPSC-derived M0 iMacs were polarized toward M1 or M2 phenotype using LPS and IFN-γ, or IL-4 and IL-13, respectively (**Figures 1A, B**). On day 31, M0, M1 and M2 iMacs were analyzed for HLA-DR and CD206 expression. M1 iMacs expressed HLA-DR, whereas no expression was detected in M0 or M2 iMacs, and M2 iMacs showed stronger expression of the CD206 marker compared with M0 and M1 iMacs (**Figure 6A**).

To assess the phagocytic capabilities of iMacs, M0, M1 and M2 iMacs were co-cultured with pHrodo Deep Red E. coli particles. Notably, after 30 min, M1 iMacs began to emit far-red fluorescence, indicating phagocytic activity (**Figure 6B**). After 2 h 30 min, phagocytic activity was observed in M0, M1, and M2 iMacs (**Figure 6C**). Taken together, these results show that M0 iMacs can be polarized towards a M1 or M2 phenotype, being well characterized by their expression of HLA-DR, CD206 and phagocytic capabilities. Using our experience on the development of this protocol, we have determined a quality control criteria and acceptance indicators by protocol stage (**Supplementary Table 2**) and we propose a set of phenotypic and functional activity criteria to define a satisfactory batch of iMacs production (**Supplementary Table 3**).

## 4 Discussion

In the present study, we established and optimized a robust, scalable, and readily adaptable to GMP- workflow for the efficient differentiation of hiPSCs into functional macrophages through hematopoietic stem cells and myeloid progenitors. By comparing coatings, media compositions, seeding densities and cell culture formats, we identified key aspects and optimized differentiation efficiency at each developmental stage. This is the first protocol differentiating clinical-grade HLA-homozygous hiPSC lines into functional iMacs combining 3D cell culture with the G-Rex platform, facilitating adaptation to large-scale upscaling in bioreactors and G-Rex system. Allogeneic cell therapies are subjected to potential allo-rejection from the host immune cells. One strategy to alleviate allo-rejection and to increase *in vivo* persistence, and therefore effectiveness, is to use HLA-homozygous donor iPSC that can be HLA-matched to the patient. This strategy has proved successful in non-human primates for the transplantation of other iPSC-derived cell types (30, 31). The work presented here shows evidence of the suitability of three iPSC lines, which are also HLA-homozygous for haplotypes of high frequency in the Spanish population and manufactured for clinical use (24).

Macrophages are becoming more relevant in cell therapy. Their phagocytic capacity, antigen-presenting activity and secretion of cytokines and chemokines make them highly interesting candidates for therapeutic applications (6, 7). CAR-Macs represent an alternative for CAR-T and CAR-NK-based therapies for the treatment of solid tumors, as they can also secrete matrix metalloproteinase and infiltrate into solid tumors, localize and persist within the TME of many cancer types, present tumor antigens to T cells, mediate phagocytosis of malignant cells, and switch from pro- to anti-tumoral inflammation (8). Furthermore, CAR-Macs have also shown potential for treating microbial and viral infections, Alzheimer’s disease, fibrosis, and atherosclerosis (12). Given all these applications, their clinical use requires a reliable and scalable source of clinical-grade macrophages. In this line, owing to their unlimited proliferation and differentiation potential, hiPSCs represent an attractive cell source for macrophage-based therapies (7). Although the first published protocols were based on 2D culture systems (19), current protocols are 3D-based (16, 17, 20). Some of the advantages of 3D protocols are that they require fewer exogenous growth factors, resemble early embryogenesis and a more physiological differentiation process, providing a niche for mesoderm induction, hematopoietic specification, and myeloid lineage commitment within the EB structure. Bioreactors also offer the potential of producing higher yields in big volume formats, highlighting the need for protocols that combine the physiological advantages of 3D differentiation with scalability and reproducibility (21, 22). Nevertheless, important challenges persist, such as shear stress associated with stirred-tank bioreactors, restricted oxygen and nutrient penetration in dense 3D cultures, the need for additional process optimization to obtain clinically relevant yields, and the reliance on highly specialized equipment (17). Notably, and in contrast to previous articles, our protocol combines the production of myeloid progenitors in 3D cell culture with subsequent differentiation to M0 and polarization into M1 and M2 iMacs using the G-Rex platform, a widely used GMP-compliant bioreactor for the manufacturing of T, NK, and hematopoietic cells.

Existing protocols describe EBs formation by seeding pluripotent stem cells as aggregates or in single cells, using non-adherent cell culture plates or AggreWell plates (25, 26). Media used include Essential 8, Essential 6, APEL2, and TeSR, with cytokine cocktails mainly containing BMP4, bFGF, VEGF, SCF, and Activin A, together with Rock inhibitor (6, 7, 17, 25–27). Here, we developed EBs by seeding hiPSCs in APEL2 medium supplemented with BMP-4, VEGF, bFGF, and Activin A during 3 days, and StemPro-34 medium with BMP-4, VEGF, bFGF, SCF, FLT3L, and IL-3 until day 10. Our protocol does not require the use AggreWell plates, simplifying the process and facilitating adaptation to large-scale upscaling in agitated tanks or vertical-wheel bioreactors. Notably, 75% of the cells were CD34^+^CD43^+^ and 55% were CD34^+^CD45^+^, consistent with a hematopoietic phenotype and supporting both the robustness and scalability of our system.

Hematopoietic stem cells are usually derived into myeloid progenitors using APEL2, StemPro-34 or X-VIVO15 medium, typically supplemented with VEGF, bFGF, SCF, IL-3, IGF-1, M-CSF, and GM-CSF for 7–10 days (6, 7, 17, 25–27). Culture coatings are often Matrigel (6) or 0.1% gelatin (27). Here, a single EB together with 25x103 CD34^+^ HSCs produced an average of 10^6^ myeloid progenitors per mL in StemPro-34 medium supplemented with human serum, M-CSF, and IL-3, using uncoated 6mw cell culture plates as a small-scale 3D cell culture system under rotation. Although we tested X-VIVO15 (data not shown), it did not perform well in our hands. By contrast, StemPro-34 supported robust myeloid progenitor differentiation in high numbers and was maintained from day 3 until the end of the differentiation process at day 31. This production of myeloid progenitors, represents a 50X of starting iPSCs, outperforming current protocols (6, 7, 22, 26). Interestingly, we found that for the best yield of MP per mL, the best combination was a mixture of EBs and a relatively low number of CD34^+^ cells. EBs alone or CD34^+^ cells alone underperformed the combination. This might reflect that both the cells within the EBs with hematopoietic potential and the secreted CD34^+^ might benefit from the other cells in the EBs, potentially endothelial lineage, mesenchymal stem cell-like and others, either by the secreted factors and/or cell-to-cell contact. Also, our data indicated that density of EBs and CD34^+^ in the cell culture is a determining factor for the yield of differentiated MPs. For up-scaling this part of the process is suspension we are planning the use of vertical wheel bioreactors -such PBS Mini Bioreactor, PBS Biotech-, which provide a gentler agitation, with considerably less sheer stress as compared to stirred tanks. The PBS Mini Bioreactor is available is growing formats of from 0,1L and 0,5L vessels to industrial sizes of several liters, and GMP versions of the vessels are available. Abundant data of the translation of small-scale multiwell plate agitation process to vertical wheel bioreactors is available in the literature for iPSC expansion and organoids formation.

In the next stage, myeloid progenitors are usually differentiated into macrophages in X-VIVO15 or RPMI medium -which in our experience performed significantly inferior to SFM StemPro™-34 plus hAB serum (data not shown)- containing principally M-CSF, and GM-CSF, and Matrigel tends to be the most common coating used (6, 7, 17, 25–27). Here, in the MP-to-iMacs step, we found that uncoated plates outperformed Matrigel, gelatin, or laminin 521, underscoring that exogenous matrix components are not required at this stage and may even impede differentiation. Furthermore, seeding density emerged as a key determinant of efficiency, with 24-well G-Rex culture system supporting higher iMac yields compared with standard culture plates. The sigmoidal relationship and subsequent decline observed between initial MP density and final iMacs in G-Rex suggests a density-dependent effect, potentially linked to available surface, nutrient gradients or autocrine/paracrine signaling. Moreover, repeated M-CSF supplementation during induction further boosted iMac output, highlighting the importance of cytokine availability for both proliferation and differentiation. Recently published protocols report the production of 4x10^7^ iMacs in 120 mL industrial stirred-tank bioreactors (28) and 1.2x10^7^ iMacs in 40 mL intermediate-scale bioreactors (22), with densities of 3.3 and 3x10^5^ iMacs per mL, respectively. In comparison, our protocol yields 1.25x10^6^ iMacs per mL (2.6x10^6^ iMacs per cm^2^), representing a 5X of seeded monocytes, using the G-Rex platform, thereby optimizing functional iMacs production per culture volume and myeloid progenitor cells. One single 500cm^2^ G-Rex bioreactor would produce up to 1.3x10^9^ iMacs, representing a clinically relevant production for the cell therapy treatment of fungal infections. Our future plans for up-scaling this protocol envision moving in progressive steps from our previous experience with 2cm^2^ in 24MW to 10cm^2^, 50cm^2^ and finally to 500cm^2^ bioreactors. G-Rex bioreactor 10cm^2^ are expected to have a direct scalability. For this up-scaling stage in G-Rex and the previous on agitation bioreactors, we are aiming for a yield comparable to the one provided in the described protocol, while maintaining the described quality criteria of phenotype and functionality.

iMacs generated under these optimized conditions displayed a typical macrophage morphology, with May-Grünwald Giemsa staining revealing large, foamy, vacuolated cytoplasm. More than 95% of resulting iMacs expressed CD14, CD86, and CD206 markers, in line with previous hiPSCs-derived macrophages (22, 25, 29). Functionally, they secreted a range of cytokines, including pro-inflammatory mediators such as TNF and IL-6, as well as IL-10, indicating immunomodulatory potential. Regarding M1 and M2 polarization, EB-derived iMacs have been reported to be prone to a higher inflammatory activity, whereas 2D-derived iMacs toward an anti-inflammatory M2-biased state (6). Here, M0 iMacs could be polarized toward M1 or M2 phenotypes with the G-Rex platform. As expected, M1 iMacs upregulated HLA-DR expression upon LPS and IFN-γ stimulation, while M0 and M2 iMacs polarized with IL-4 and IL-13 did not. The ability to recapitulate polarization states is a hallmark of primary human macrophages and demonstrates the functional relevance of the iMacs produced. Furthermore, phagocytic assays with pHrodo E. coli bioparticles confirmed the acquisition of a key macrophage function. Altogether, our protocol has demonstrated the capacity to produce highly pure and functional macrophages that recapitulate the fundamental characteristics of primary monocyte-derived macrophages.

Together, these findings demonstrate that hiPSC-derived macrophages can be generated in large numbers under defined conditions, with efficiency strongly dependent on culture format, seeding strategy, and cytokine management. The use of G-Rex culture system for MP-to-iMac differentiation represents a scalable approach, potentially enabling clinical translation where large macrophage numbers are required. Moreover, the ability to produce polarized macrophage subsets with preserved functionality supports the utility of this system for modeling immune responses, studying disease mechanisms, and developing macrophage-based therapies.

Limitations of this study include the reliance on EB-based induction, which, although robust, may introduce heterogeneity. Another limitation is the use of human serum during the hematopoietic induction step, due to its undefined nature, although this can be compatible with GMP standards if the serum is sourced, processed, and documented in accordance with strict regulatory requirements. Future work should also compare transcriptional and functional profiles of hiPSC-derived iMacs with primary monocyte-derived macrophages across multiple donors to assess equivalence. Additionally, long-term functionality, antigen presentation capacity, genomic integrity and *in vivo* behavior remain to be fully explored.

## 5 Conclusion

We present a robust and scalable workflow for the differentiation of hiPSCs into functional macrophages via HSCs and MPs, optimized across substrate, cytokine, and culture system parameters. EB-based suspension culture achieved the highest yields, and M-CSF remained essential throughout the process. Notably, macrophage differentiation was most efficient under matrix-free conditions, and seeding density was identified as a key determinant of expansion. G-Rex culture system showed a maintained yield in the MP to Mac step with high seeding densities, which facilitates the production in reduced surfaces, suitable for large scale productions. The protocol yields iMacs with the expected phenotype, cytokine secretion, polarization capacity, and phagocytic activity. Together, these results provide a strong foundation for the use of hiPSCs-derived macrophages in immunological research, disease modeling, and, more importantly, potential cell-based therapies.

## Data Availability

The original contributions presented in the study are included in the article/**Supplementary Material**. Further inquiries can be directed to the corresponding author.

## Supplementary material

Supplementary Figure 1 Comparison between macrophages derived from primary peripheral blood monocytes and iPSC-derived macrophages. A Representative plots of the gating strategy used during flow cytometry analysis: exclusion of cellular debris using FSC-A and SSC-A; selection of single cells using FSC-A and FSC-H; and identification of live cells using SSC-A and ViaKrome 405. B Overlay of MFI histograms comparing CD14, CD86 and CD206 expression between PB-Macs and iMacs.

## References

[1] Ding T Du Y Yang B Tian W Li J Xie J . Comprehensive review of macrophage models: primary cells and immortalized lines across species. Front Immunol. (2025) 16:1640935. doi: 10.3389/fimmu.2025.1640935, PMID: 40909285 PMC12404969

[2] Lazarov T Juarez-Carreño S Cox N Geissmann F . Physiology and diseases of tissue-resident macrophages. Nature. (2023) 618:698–707. doi: 10.1038/s41586-023-06002-x, PMID: 37344646 PMC10649266

[3] Luo M Zhao F Cheng H Su M Wang Y . Macrophage polarization: an important role in inflammatory diseases. Front Immunol. (2024) 15:1352946. doi: 10.3389/fimmu.2024.1352946, PMID: 38660308 PMC11039887

[4] Yang S Wang Y Jia J Fang Y Yang Y Yuan W . Advances in engineered macrophages: A new frontier in cancer immunotherapy. Cell Death Dis. (2024) 15:238. doi: 10.1038/s41419-024-06616-7, PMID: 38561367 PMC10985090

[5] Karagiannis P Takahashi K Saito M Yoshida Y Okita K Watanabe A . Induced pluripotent stem cells and their use in human models of disease and development. Physiol Rev. (2019) 99:79–114. doi: 10.1152/physrev.00039.2017, PMID: 30328784

[6] Zhang L Tian L Dai X Yu H Wang J Lei A . Pluripotent stem cell-derived CAR-macrophage cells with antigen-dependent anti-cancer cell functions. J Hematol Oncol. (2020) 13:153. doi: 10.1186/s13045-020-00983-2, PMID: 33176869 PMC7656711

[7] Lei A Yu H Lu S Lu H Ding X Tan T . A second-generation M1-polarized CAR macrophage with antitumor efficacy. Nat Immunol. (2024) 25:102–16. doi: 10.1038/s41590-023-01687-8, PMID: 38012418

[8] Lu J Ma Y Li Q Xu Y Xue Y Xu S . CAR Macrophages: a promising novel immunotherapy for solid tumors and beyond. biomark Res. (2024) 12:86. doi: 10.1186/s40364-024-00637-2, PMID: 39175095 PMC11342599

[9] Peng L Sferruzza G Yang L Zhou L Chen S . CAR-T and CAR-NK as cellular cancer immunotherapy for solid tumors. Cell Mol Immunol. (2024) 21:1089–108. doi: 10.1038/s41423-024-01207-0, PMID: 39134804 PMC11442786

[10] Liu Y Xiao L Yang M Chen X Liu H Wang Q . CAR-armored-cell therapy in solid tumor treatment. J Transl Med. (2024) 22:1076. doi: 10.1186/s12967-024-05903-3, PMID: 39609705 PMC11603843

[11] Pan K Farrukh H Chittepu VCSR Xu H Pan C Zhu Z . CAR race to cancer immunotherapy: from CAR T, CAR NK to CAR macrophage therapy. J Exp Clin Cancer Res. (2022) 41:119. doi: 10.1186/s13046-022-02327-z, PMID: 35361234 PMC8969382

[12] Chen Y Xin Q Zhu M Qiu J Luo Y Li R . Exploring CAR-macrophages in non-tumor diseases: Therapeutic potential beyond cancer. J Adv Res. (2025) 77:481–96. doi: 10.1016/j.jare.2025.01.004, PMID: 39756574 PMC12627343

[13] Abdin SM Paasch D Kloos A Oliveira MC Jang M-S Ackermann M . Scalable generation of functional human iPSC-derived CAR-macrophages that efficiently eradicate CD19-positive leukemia. J Immunother Cancer. (2023) 11:e007705. doi: 10.1136/jitc-2023-007705, PMID: 38135346 PMC10749073

[14] Takahashi K Tanabe K Ohnuki M Narita M Ichisaka T Tomoda K . Induction of pluripotent stem cells from adult human fibroblasts by defined factors. Cell. (2007) 131:861–72. doi: 10.1016/j.cell.2007.11.019, PMID: 18035408

[15] Martin KE Hammer Q Perica K Sadelain M Malmberg K-J . Engineering immune-evasive allogeneic cellular immunotherapies. Nat Rev Immunol. (2024) 24:680–93. doi: 10.1038/s41577-024-01022-8, PMID: 38658708

[16] van Wilgenburg B Browne C Vowles J Cowley SA . Efficient, long term production of monocyte-derived macrophages from human pluripotent stem cells under partly-defined and fully-defined conditions. PLoS One. (2013) 8:e71098. doi: 10.1371/journal.pone.0071098, PMID: 23951090 PMC3741356

[17] Lachmann N Ackermann M Frenzel E Liebhaber S Brennig S Happle C . Large-scale hematopoietic differentiation of human induced pluripotent stem cells provides granulocytes or macrophages for cell replacement therapies. Stem Cell Rep. (2015) 4:282–96. doi: 10.1016/j.stemcr.2015.01.005, PMID: 25680479 PMC4325194

[18] Ackermann M Kempf H Hetzel M Hesse C Hashtchin AR Brinkert K . Bioreactor-based mass production of human iPSC-derived macrophages enables immunotherapies against bacterial airway infections. Nat Commun. (2018) 9:5088. doi: 10.1038/s41467-018-07570-7, PMID: 30504915 PMC6269475

[19] Cao X Yakala GK van den Hil FE Cochrane A Mummery CL Orlova VV . Differentiation and functional comparison of monocytes and macrophages from hiPSCs with peripheral blood derivatives. Stem Cell Rep. (2019) 12:1282–97. doi: 10.1016/j.stemcr.2019.05.003, PMID: 31189095 PMC6565887

[20] Nenasheva T Gerasimova T Serdyuk Y Grigor’eva E Kosmiadi G Nikolaev A . Macrophages derived from human induced pluripotent stem cells are low-activated “Naïve-like” Cells capable of restricting mycobacteria growth. Front Immunol. (2020) 11:1016. doi: 10.3389/fimmu.2020.01016, PMID: 32582159 PMC7287118

[21] Hai Q Bazeley P Han J Brubaker G Powers J Diaz-Montero CM . Optimized method to generate well-characterized macrophages from induced pluripotent stem cells. Biomedicines. (2025) 13:99. doi: 10.3390/biomedicines13010099, PMID: 39857683 PMC11762477

[22] Ackermann M Saleh F Abdin SM Rafiei Hashtchin A Gensch I Golgath J . Standardized generation of human iPSC-derived hematopoietic organoids and macrophages utilizing a benchtop bioreactor platform under fully defined conditions. Stem Cell Res Ther. (2024) 15:171. doi: 10.1186/s13287-024-03785-2, PMID: 38886860 PMC11184717

[23] Bajgain P Mucharla R Wilson J Welch D Anurathapan U Liang B . Optimizing the production of suspension cells using the G-Rex “M” series. Mol Ther - Methods Clin Dev. (2014) 1:14015. doi: 10.1038/mtm.2014.15, PMID: 26015959 PMC4362380

[24] Kuebler B Alvarez-Palomo B Aran B Castaño J Rodriguez L Raya A . Generation of a bank of clinical-grade, HLA-homozygous iPSC lines with high coverage of the Spanish population. Stem Cell Res Ther. (2023) 14:366. doi: 10.1186/s13287-023-03576-1, PMID: 38093328 PMC10720139

[25] Vaughan-Jackson A Stodolak S Ebrahimi KH Browne C Reardon PK Pires E . Differentiation of human induced pluripotent stem cells to authentic macrophages using a defined, serum-free, open-source medium. Stem Cell Rep. (2021) 16:1735–48. doi: 10.1016/j.stemcr.2021.05.018, PMID: 34171284 PMC8282471

[26] Li S Song L Zhang Y Zhan Z Yang Y Yu L . Optimizing the method for differentiation of macrophages from human induced pluripotent stem cells. Stem Cells Int. (2022) 2022:1–13. doi: 10.1155/2022/6593403, PMID: 35283995 PMC8913134

[27] Pouyanfard S Meshgin N Cruz LS Diggle K Hashemi H Pham TV . Human induced pluripotent stem cell-derived macrophages ameliorate liver fibrosis. Stem Cells. (2021) 39:1701–17. doi: 10.1002/stem.3449, PMID: 34460131

[28] Ackermann M Rafiei Hashtchin A Manstein F Carvalho Oliveira M Kempf H Zweigerdt R . Continuous human iPSC-macrophage mass production by suspension culture in stirred tank bioreactors. Nat Protoc. (2022) 17:513–39. doi: 10.1038/s41596-021-00654-7, PMID: 35039668 PMC7612500

[29] Munn C Burton S Dickerson S Bakshy K Strouse A Rajesh D . Generation of cryopreserved macrophages from normal and genetically engineered human pluripotent stem cells for disease modelling. PLoS One. (2021) 16:e0250107. doi: 10.1371/journal.pone.0250107, PMID: 33886609 PMC8061979

[30] Sugita S Iwasaki Y Makabe K Kamao H Mandai M Shiina T . Successful transplantation of retinal pigment epithelial cells from MHC homozygote iPSCs in MHC-matched models. Stem Cell Rep. (2016) 7:635–48. doi: 10.1016/j.stemcr.2016.08.010, PMID: 27641649 PMC5063629

[31] Morizane A Kikuchi T Hayashi T Mizuma H Takara S Doi H . MHC matching improves engraftment of iPSC-derived neurons in non-human primates. Nat Commun. (2017) 8:385. doi: 10.1038/s41467-017-00926-5, PMID: 28855509 PMC5577234

