# Supplementary material for: Robust, scalable and xeno-free protocol for differentiating human induced pluripotent stem cells into functional macrophages
Source: Front Immunol. 2026 Jan 12;16:1719452. doi: 10.3389/fimmu.2025.1719452 (PMC12833622; doi:10.3389/fimmu.2025.1719452)
Supplement: Supplementary file 1 [file Table1.docx]

**Supplementary Table 1. Resources Table**

| **REAGENT or RESOURCE** | **SOURCE** | **CATALOGUE NUMBER** | **GRADE** | **AVAILABILITY GMP GRADE** |
| --- | --- | --- | --- | --- |
| **HLA-homozygous hiPS Cell Lines** | | | | |
| HL4 | iPS-PANIA cell line collection | Hz 1 8 3 CBiPS4 Sv4F F6 | Clinical-Grade |  |
| HL6 | iPS-PANIA cell line collection | Hz 33 14 1 CBiPS6 Sv4F H6 | Clinical-Grade |  |
| HL7 | iPS-PANIA cell line collection | Hz 24 7 15 CBiPS7 Sv4F I12 | Clinical-Grade |  |
| **Reagents for differentiation protocol** | | | | |
| TeSR™-AOF | Stemcell Technologies | 100-0401 | GMP | YES |
| CTS™ Versene™ | Thermo Fisher Scientific | A4239101 | CTS | YES |
| PBS -Ca^2+^ / -Mg^2+^ | Corning | 21-031-CV | RUO | YES |
| PBS +Ca^2+^ / +Mg^2+^ | Cytiva | SH30264.01 | RUO | YES |
| Y-27632 dihydrochloride | Sigma | Y0503 | RUO | YES |
| StemPro™ Accutase™ | Thermo Fisher Scientific | A1110501 | GMP | YES |
| STEMdiff™ APEL™2 Medium | Stemcell Technologies | 05270 | RUO | YES |
| SFM StemPro™-34 | Thermo Fisher Scientific | 10639-011 | GMP | YES |
| GlutaMAX™ (100X) | Thermo Fisher Scientific | 35050038 | GMP | YES |
| MEM Non-Essential Amino Acids Solution (100X) | Thermo Fisher Scientific | 11140050 | GMP | YES |
| Insulin-Transferrin-Selenium-Ethanolamine (ITS -X) (100X) | Thermo Fisher Scientific | 51500056 | GMP | YES |
| 2-Mercaptoethanol (50 mM) | Thermo Fisher Scientific | 31350010 | GMP | YES |
| Human AB serum | Banc de Sang i Teixits | D5144V00 | Clinical-grade | YES |
| CellAdhere™ Laminin-521 | Stemcell Technologies | 200-0117 | RUO | YES |
| rh BMP-4 | R&D Systems | 314-BP-010 | RUO | YES |
| rh VEGF-165 | Stemcell Technologies | 78159.1 | RUO | YES |
| rh bFGF | Stemcell Technologies | 78134.1 | RUO | YES |
| rh Activin A | R&D Systems | 338-GMP-010 | GMP | YES |
| rh SCF | Thermo Fisher Scientific | AF-300-07 | RUO | YES |
| rh FLT3L | Thermo Fisher Scientific | AF-300-19 | RUO | YES |
| rh IL-3 | Miltenyi Biotec | 130-095-070 | RUO | YES |
| L-Ascorbic acid | Sigma-Aldrich | A4403-100MG | RUO | YES |
| rh M-CSF | Thermo Fisher Scientific | 300-25-50UG | RUO | YES |
