# Supplementary material for: Robust, scalable and xeno-free protocol for differentiating human induced pluripotent stem cells into functional macrophages
Source: Front Immunol. 2026 Jan 12;16:1719452. doi: 10.3389/fimmu.2025.1719452 (PMC12833622; doi:10.3389/fimmu.2025.1719452)
Supplement: Supplementary file 2 [file Table2.docx]

**Supplementary Table 2. Quality control criteria and indicators by protocol stage.**

| **Stage** | **QA/QC Parameter** | **Acceptance Criteria** |
| --- | --- | --- |
| Human induced pluripotent stem cell cultivation | hiPSC cell morphology | (i) Adhered, compact colonies, (ii) with a high nucleus-to-cytoplasm ratio, and (iii) no signs of spontaneous differentiation. |
|  | hiPSC proliferation | (i) Passaging twice a week with a dilution 1/8 – 1/10 and (ii) having a confluency the day of passage between 80-90%. |
|  | Mycoplasma | Negative by PCR. |
|  | Sterility | Differentiation maintained in antibiotic-free medium and (i) absence of visible contamination. |
| Differentiation of iPSCs into hematopoietic stem cells | Starting material | Differentiation of hiPSCs initiated when (i) confluency ≥80%, (ii) stable colony morphology, (iii) absence of spontaneous differentiation, and (iv) viability of undifferentiated single-cell hiPSCs ≥95% (using Tripan Blue). |
|  | hiPSCs pluripotency | EB formation at day 3 (i) measuring >300µm, (ii) having spherical morphology and (iii) showing clear boundaries. |
|  | Differentiation | (i) ≥5x10^5^/mL secreted cells, (ii) viability ≥95%, and (iii) ≥50% CD34+CD43+ (measured using Hematopoietic stem cells flow cytometry panel). |
|  | Sterility | Differentiation maintained in antibiotic-free medium and (i) absence of visible contamination. |
| Differentiation of hematopoietic stem cells into myeloid progenitors | Starting material | Differentiation of CD34-producing EBs initiated when (i) ≥5x10^5^/mL secreted cells, (ii) viability ≥95%, and (iii) ≥50% CD34+CD43+ (measured using Hematopoietic stem cells flow cytometry panel). |
|  | Differentiation | (i) ≥3x10^6^/mL secreted cells, (ii) viability ≥95%, and (iii) ≥50% CD45+CD14+ (measured using Myeloid progenitor cells flow cytometry panel). |
|  | Sterility | Differentiation maintained in antibiotic-free medium and (i) absence of visible contamination. |
| Differentiation of myeloid progenitors into M0 iMacs | Starting material | Differentiation of myeloid progenitors initiated when (i) ≥3x10^6^/mL secreted cells, (ii) viability ≥95%, and (iii) ≥50% CD45+CD14+ (measured using Myeloid progenitor cells flow cytometry panel). |
|  | Differentiation | (i) ≥3X fold, (ii) viability ≥95%, and (iii) 85% CD14+CD86+CD206+ (measured using iMacs flow cytometry panel). |
|  | Morphology | (i) May-Grünwald Giemsa staining revealing large, foamy, vacuolated cytoplasm. |
|  | Phagocytic capacity | (i) Internalization of pHrodo E. coli BioParticles after 2h at 37 ºC in ≥95% iMacs (measured using pHrodo flow cytometry assay). |
|  | Cytokine production | (i) IL-6, (ii) IL-10, and (iii) Granzyme-B (measured using single cytokine ELISAs or multiplex). |
|  | Sterility | Differentiation maintained in antibiotic-free medium and (i) absence of visible contamination. |
| Polarization of M0 iMacs towards M1 or M2 phenotype | Starting material | (i) Viability ≥95%, (ii) ≥3X fold, and (iii) 85% CD14+CD86+CD206+ (measured using iMacs flow cytometry panel). |
|  | Polarization | (i) Viability ≥90%, and (ii) increase in HLA-DR or CD206 markers (for M1 or M2, respectively; measured using iMacs flow cytometry panel). |
|  | Morphology | (i) Elongated, spindle-shaped appearance for M1; and large, round, and flat shape with an amoeboid appearance for M2 iMacs |
|  | Phagocytic capacity | (i) Internalization of pHrodo E. coli BioParticles after 2h at 37 ºC in ≥95% iMacs (measured using pHrodo flow cytometry assay). |
|  | Sterility | Differentiation maintained in antibiotic-free medium and (i) absence of visible contamination. |

iMacs, human induced pluripotent stem cell-derived macrophages.
