# Supplementary material for: Robust, scalable and xeno-free protocol for differentiating human induced pluripotent stem cells into functional macrophages
Source: Front Immunol. 2026 Jan 12;16:1719452. doi: 10.3389/fimmu.2025.1719452 (PMC12833622; doi:10.3389/fimmu.2025.1719452)
Supplement: Supplementary file 3 [file Table3.docx]

**Supplementary Table 3. Minimum Quality Requirements for iMacs production.**

| **Stage** | **QA/QC Parameter** | **Acceptance Criteria** |
| --- | --- | --- |
| Final product: M0 iMacs | Viability | ≥85% (measured using iMacs flow cytometry panel). |
|  | Phenotype | 85% CD14+CD86+CD206+ (measured using iMacs flow cytometry panel). |
|  | Morphology | Large, foamy, and vacuolated cytoplasm (by optical inspection of May-Grünwald Giemsa preparations). |
|  | Phagocytic capacity | Internalization of pHrodo E. coli BioParticles after 2h at 37 ºC in ≥85% iMacs (measured using pHrodo flow cytometry assay). |
|  | Cytokine production | ≥10 pg/mL IL-6, IL-10, and Granzyme-B (measured using single cytokine ELISAs or multiplex). |
|  | Polarization capability | Morphological changes, and increase in HLA-DR or CD206 markers (for M1 or M2, respectively; measured using iMacs flow cytometry panel). |
|  | Sterility | Absence of visible contamination. |

iMacs, human induced pluripotent stem cell-derived macrophages.
