# Supplementary figures and images for: Robust, scalable and xeno-free protocol for differentiating human induced pluripotent stem cells into functional macrophages
Source: Front Immunol. 2026 Jan 12;16:1719452. doi: 10.3389/fimmu.2025.1719452 (PMC12833622; doi:10.3389/fimmu.2025.1719452)

## Slide 1
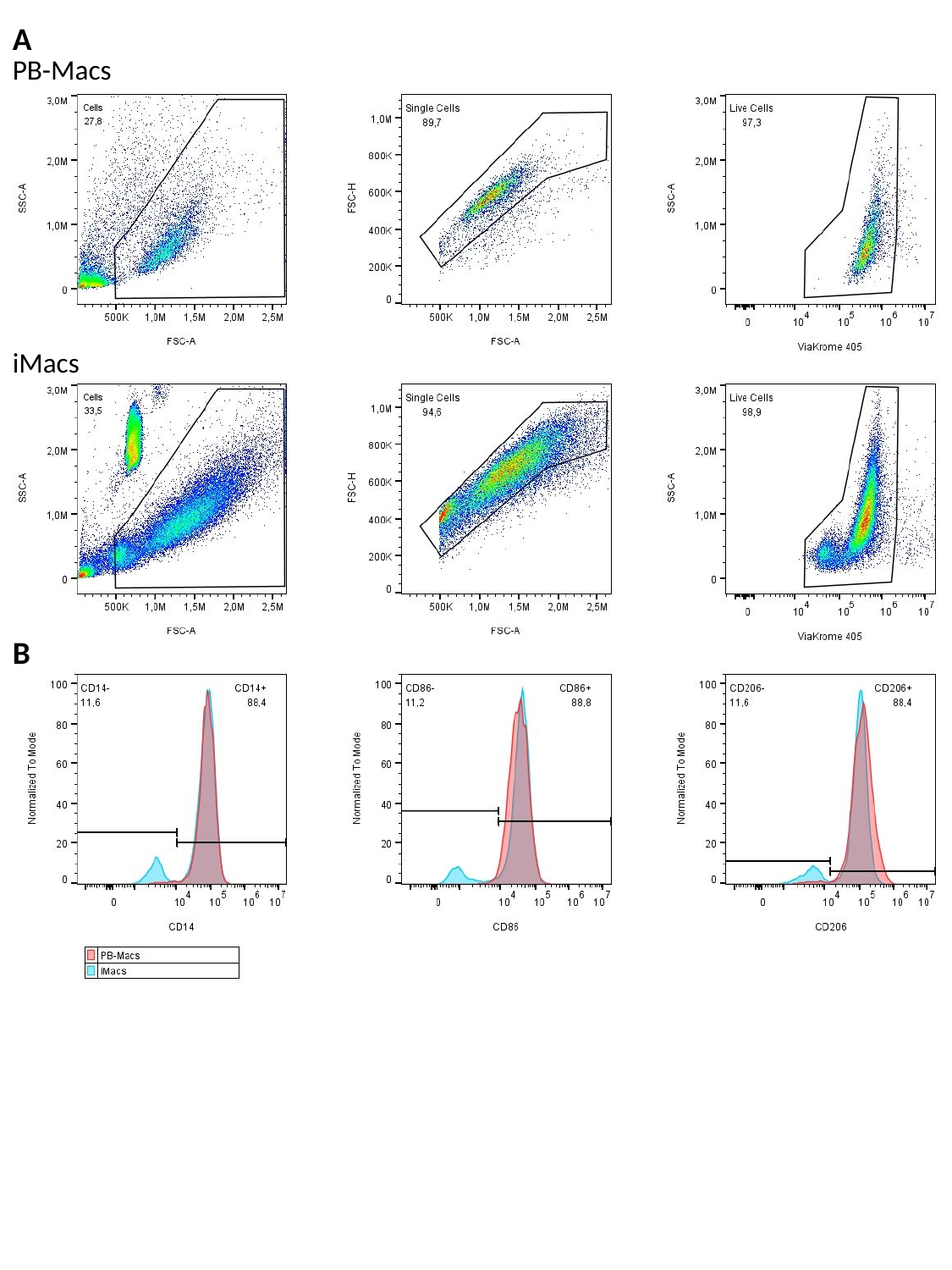

A
PB-Macs
iMacs
B

Supplement: Supplementary Figure 1 — Comparison between macrophages derived from primary peripheral blood monocytes and iPSC-derived macrophages. A Representative plots of the gating strategy used during flow cytometry analysis: exclusion of cellular debris using FSC-A and SSC-A; selection of single cells using FSC-A and FSC-H; and identification of live cells using SSC-A and ViaKrome 405. B Overlay of MFI histograms comparing CD14, CD86 and CD206 expression between PB-Macs and iMacs. [file Presentation1.pptx]
